# Tumour Stroma Ratio Assessment Using Digital Image Analysis Predicts Survival in Triple Negative and Luminal Breast Cancer

**DOI:** 10.3390/cancers12123749

**Published:** 2020-12-13

**Authors:** Ewan KA Millar, Lois H. Browne, Julia Beretov, Kirsty Lee, Jodi Lynch, Alexander Swarbrick, Peter H. Graham

**Affiliations:** 1 Department of Anatomical Pathology, New South Wales Health Pathology, St George Hospital, Kogarah, NSW 2217, Australia; julia.beretov@health.nsw.gov.au; 2 St George & Sutherland Clinical School, University of New South Wales Sydney, Kensington, NSW 2052, Australia; peter.graham@health.nsw.gov.au; 3 Faculty of Medicine & Health Sciences, Sydney Western University Campbelltown, Campbelltown, NSW 2560, Australia; 4 Cancer Care Centre, St George Hospital, Kogarah, NSW 2217, Australia; lois.browne@health.nsw.gov.au (L.H.B.); jodi.lynch@health.nsw.gov.au (J.L.); 5 Department of Clinical Oncology, Prince of Wales Hospital, Chinese University of Hong Kong, Shatin, N.T., Hong Kong; kirstylee@clo.cuhk.edu.hk; 6 Garvan Institute of Medical Research, 370 Victoria Street, Darlinghurst, NSW 2010, Australia; a.swarbrick@garvan.org.au; 7 Kinghorn Cancer Centre, 370 Victoria Street, Darlinghurst, Sydney, NSW 2010, Australia; 8 St Vincent’s Clinical School, Darlinghurst, University of New South Wales, Sydney, NSW 2010, Australia

**Keywords:** breast cancer, biomarker, image analysis, machine learning, prognosis

## Abstract

**Simple Summary:**

Tumour stroma is known to predict outcome and play an important role in the growth and spread of solid tumours and their response to therapy. In breast cancer, there is evidence that the tumour stroma ratio (TSR) can predict outcome in aggressive triple negative breast cancer, but its value for the more common hormone receptor positive breast cancer is unclear. Using computerised image analysis and machine learning algorithms, we show that TSR is an important factor in predicting outcome for triple negative disease and hormone receptor positive cancer. However, its influence on good or poor outcome appears to depend on tumour type and the relative predominance of the stromal component. By better understanding the role of the tumour stroma in cancer growth, and its response to treatment, this study may help support the role of TSR as a new prognostic marker for breast cancer to guide clinical decision making.

**Abstract:**

We aimed to determine the clinical significance of tumour stroma ratio (TSR) in luminal and triple negative breast cancer (TNBC) using digital image analysis and machine learning algorithms. Automated image analysis using QuPath software was applied to a cohort of 647 breast cancer patients (403 luminal and 244 TNBC) using digital H&E images of tissue microarrays (TMAs). Kaplan–Meier and Cox proportional hazards were used to ascertain relationships with overall survival (OS) and breast cancer specific survival (BCSS). For TNBC, low TSR (high stroma) was associated with poor prognosis for both OS (HR 1.9, CI 1.1–3.3, *p* = 0.021) and BCSS (HR 2.6, HR 1.3–5.4, *p* = 0.007) in multivariate models, independent of age, size, grade, sTILs, lymph nodal status and chemotherapy. However, for luminal tumours, low TSR (high stroma) was associated with a favourable prognosis in MVA for OS (HR 0.6, CI 0.4–0.8, *p* = 0.001) but not for BCSS. TSR is a prognostic factor of most significance in TNBC, but also in luminal breast cancer, and can be reliably assessed using quantitative image analysis of TMAs. Further investigation into the contribution of tumour subtype stromal phenotype may further refine these findings.

## 1. Introduction

Breast cancer is the most common cancer in women and the most common cause of cancer deaths worldwide with an estimated 2.1 million cases diagnosed and more than 620,000 deaths globally in 2018 [1]. Routine prognostic and predictive biomarkers to stratify patient risk, including estrogen receptor (ER), progesterone receptor (PR), human epidermal growth factor receptor-2 (HER2) and Ki67, have remained largely unchanged in almost two decades. The tumour microenvironment (TME), which is actively involved in growth promotion and progression in solid tumours [2,3] has now been actively included in the search for new meaningful biomarkers. The tumour stroma contains cancer-associated fibroblasts (CAFs), known to be involved in cellular crosstalk, the induction of local immunosuppression and resistance to chemotherapy in triple negative breast cancer (TNBC) and endocrine therapy in ER+ breast cancer [4,5,6,7].

Tumour stroma ratio (TSR) is a prognostic biomarker of importance in most solid tumours (reviewed in detail [8]) with high stromal content overall tending to be associated with a poorer prognosis. In breast cancer several studies have demonstrated an association of poor outcome with high stroma in TNBC, but there have been some conflicting results for ER+ disease (reviewed in detail in [9]). High stroma has been associated with poor prognosis in several studies [10,11,12] with favourable outcome in one study [13]. Single cell transcriptomic studies have identified the diverse phenotype of cells present in the TME, which include immune cells, cancer associated fibroblasts (CAFs), endothelial cells and pericytes [6,14]. There is increasing evidence to suggest that CAFs may provide a specialised niche for cancer stem cells and influence responsiveness to chemotherapy in TNBC [5,15,16] and endocrine therapy in ER+ disease [17,18], supported by more recent single cell sequencing studies identifying specific subtypes of CAFs in mouse and human breast tumours [14,19]. Therefore, further data to support the rationale for including TSR as a prognostic variable in breast cancer, with clinical significance, especially for TNBC which has no other existing biomarkers, would be of value in patient risk stratification. The consideration of TSR as a new breast cancer biomarker could be supported through its inclusion in prospective clinical trials, such is the case in colorectal cancer [20].

Most studies of TSR to date have been employed by one established group of experienced investigators using visual assessment of TSR in whole tumour slides, using a predefined cut point of 50% stroma to delineate a high or low stromal content tumour [12,21,22]. Another group of investigators used a more complex point counting method of visual assessment in two studies [13,23]. The aim of this study was to use automated quantitative image analysis algorithms to assess TSR and determine its clinical significance in luminal and TNBC. A similar approach using deep learning convolutional neural network (CNN) artificial intelligence (AI) in colorectal carcinoma showed improved predictive power over expert human visual assessment [24].

Utilising a high throughput approach, we assessed digital images of H&E stained tissue microarrays (TMAs) to calculate quantitative measurements of tumour and stromal areas in a cohort of 647 luminal and TNBC patients. This approach offers the potential to overcome possible subjective visual assessment and, more significantly, eliminate the onerous time and workflow bottleneck required by visual scoring performed by an experienced Pathologist. This pipeline efficiently allows data to be generated in hours rather than months. Improved image analysis software includes many easy-to-use platforms, commercially available or open source free to access, such as QuPath [25]. With rapidly expanding expertise in deep learning AI, such algorithms are becoming more common research tools to assess more complex visual features within pathology images of potential clinical significance [24]. However, before any new biomarker may be used clinically, rigorous testing and validation in independent cohorts with large numbers of patient samples is required. High-throughput digital image analysis of TMA cores provides the methodology to provide this data. In this study, we aimed to determine the prognostic significance of TSR in breast cancer using computerized image analysis and machine learning algorithms.

## 2. Results

### 2.1. Tumour Stroma Ratios (TSR)

**Figure 1:**
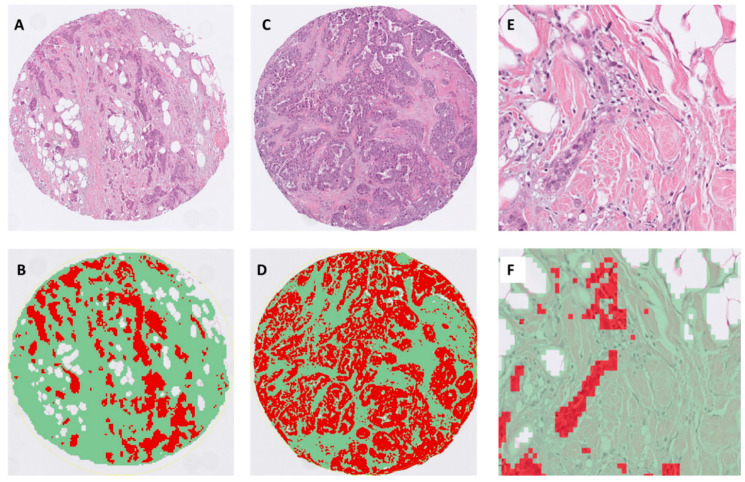
Representative matched H&E and segmented images of tumour epithelium (red), stroma (green) and fat (white) using the pixel classifier algorithm in QuPath. The images display examples of invasive ductal carcinoma with (**A**,**B**): low tumour stroma ratio (TSR) (i.e., stroma high, TSR: 0.35, ×100), (**C**,**D**) intermediate TSR (roughly equivalent volumes of epithelium and stroma, TSR: 0.9, ×100); (**E**,**F**): high power of invasive ductal carcinoma with adjacent fat with a mild infiltrate of stromal tumour infiltrating lymphocytes (TILs) (×400).

Examples of representative TSR images generated by the pixel classifier in QuPath is presented in Figure 1. The distribution of TSR versus stromal percentage for all tumours and the distribution of TSR values between luminal and TNBC tumours is presented in Figure S1A,B. These data highlight the inverse relationship between TSR and stromal percentage, i.e., high TSR is equivalent to a low % stroma and vice versa (Figure S1A). The Boost cohort was first assessed in a preliminary analysis with a median TSR value of 0.84. Stratifying the whole cohort of luminal ER+ or TNBC cases by this value was not significant for OS. We next analysed the distribution of TSR scores, which showed a significant difference between luminal and TNBC tumours (Figure S1B, *p* < 0.001) and between histological type and grade for luminal tumours (Figure S1D and Table S1, *p* < 0.001), but was nonsignificant for TNBC (Figure S1C). We then used the median values for TSR to specifically define high or low TSR, according to the molecular subtype of the tumour, to further explore the relationship with outcome (luminal tumour median TSR 0.74, TNBC 2.0). We then retained the TNBC cut point, derived and applied this to the whole TNBC cohort, as a validation procedure.

### Association of TSR with Clinico-Pathological Features

There is a highly significant correlation between TSR with luminal ER+ histological tumour type and grade (both *p* < 0.001), but not for TNBC. In luminal tumours, the TSR value increased in a step-like fashion from lobular to tubular to invasive ductal NOS (*p* < 0.001, Figure S1D, with a similar trend for grade (mean TSR grade 1, 0.84; grade 2, 1.2; grade 3, 2.3). TNBC TSR correlated with age (*p* = 0.006) and sTILs (*p* = 0.010) only. Luminal TSR correlated with age (*p* = 0.043), size (*p* = 0.021), grade (*p* < 0.001), lymph nodal status (*p* = 0.036) and sTILs (*p* < 0.001; Table S1).

### 2.2. Survival Analyses

#### 2.2.1. Triple Negative Breast Cancer

**Figure 2:**
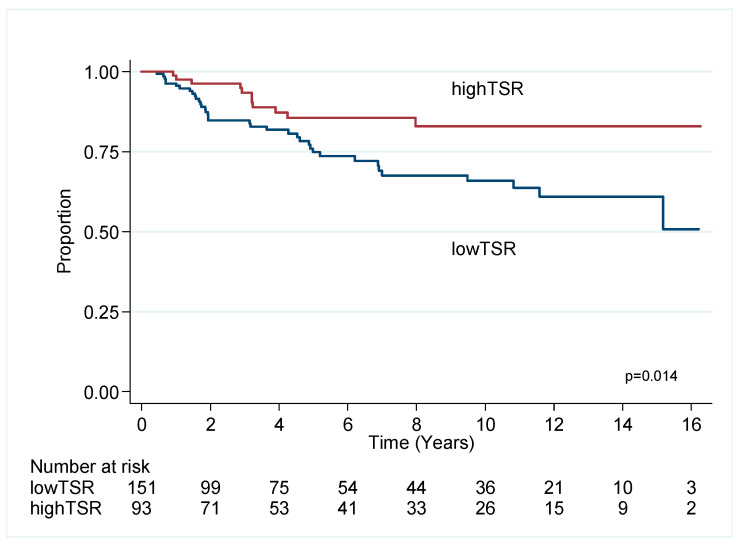
Kaplan–Meier survival estimates for breast cancer specific survival in TNBC.

Cox univariate and multivariable (MVA) analyses demonstrated that low TSR (i.e., high stroma) predicts poor outcome for OS (HR 1.90, CI 1.10–3.29, *p* = 0.021) and BCSS (HR 2.64, 1.31–5.35, *p* = 0.007) in MVA models after consideration of age, size, grade, sTILs, lymph nodal status and chemotherapy (Table 1, Figure 2).

#### 2.2.2. Combined TSR and TILs Impact on Outcome in TNBC

**Figure 3:**
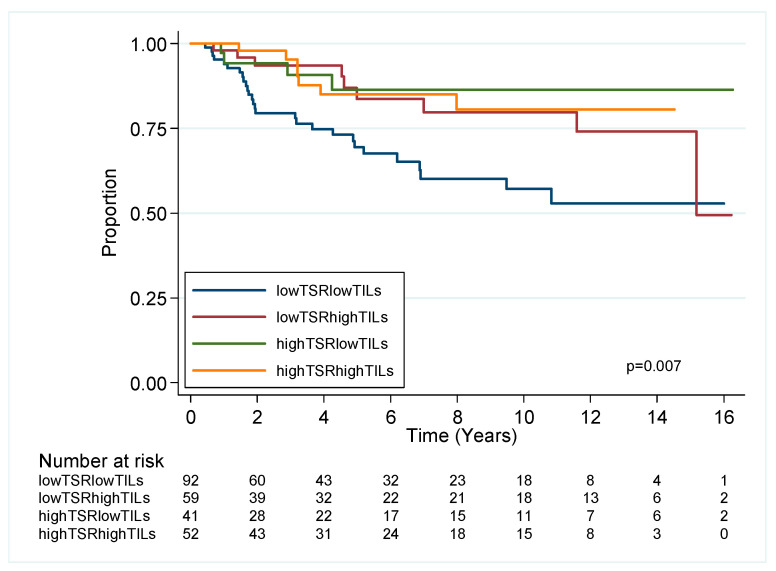
Kaplan–Meier survival estimates for breast cancer specific survival in TNBC stratified by TSR and TILs.

Given the relationship of TILS with survival in TNBC, we used the cut point of ≤30% or >30% to define low or high TILs in keeping with recent studies [26,27] which demonstrated high TILs associated with improved prognosis for OS/BCSS univariate analysis. A positive correlation of high TSR and high TILs (p = 0.01, Table S1), directed us to next assess TNBC outcome for OS and BCSS, dependent on combined TSR and sTILs status (Figure 3). This stratified the TNBC cohort into 4 groups, with the best prognostic group (high TSR/high TILs) having approximately a 69% reduction in risk of death from breast cancer compared to the worst prognostic group (low TSR low TILs), which retained independent prognostic significance in MVA for OS and BCSS (Table S2).

#### 2.2.3. Chemotherapy and TSR in TNBC

TSR did not predict outcome for OS or BCSS for those TNBC patients treated with chemotherapy (*p* = 0.268, *p* = 0.141, respectively), suggesting its value is as a prognostic rather than a predictive biomarker.

### 2.3. Luminal Breast Cancer

**Figure 4:**
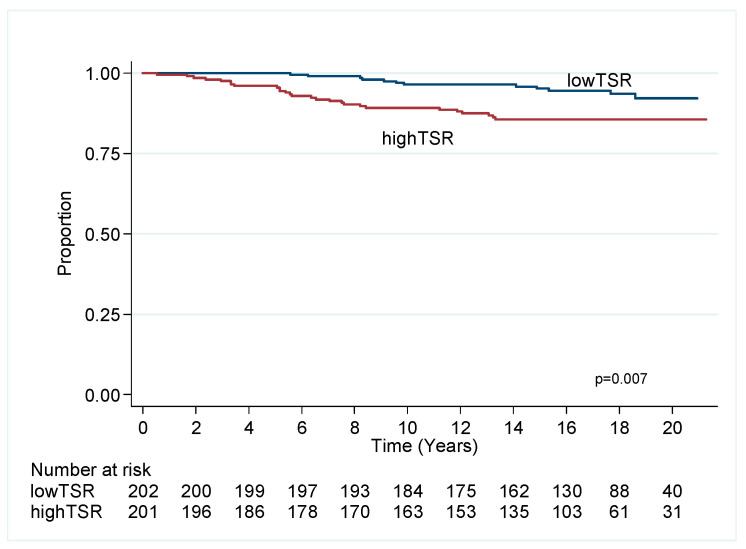
Kaplan–Meier plot of TSR for luminal ER+ cancer, breast cancer specific survival.

Cox univariate and multivariable analyses (Table 2) demonstrate that low TSR (i.e., high stroma) predicts favourable outcome for OS (HR 0.56, CI 0.4–0.77, *p* = 0.001) in MVA in a model independent of age, size, grade, sTILs, lymph nodal status, endocrine therapy and chemotherapy. Low TSR is significant in univariate analysis for BCSS (Figure 4) but not in MVA.

#### Endocrine Therapy and TSR

High TSR predicted poor outcome in luminal cancers treated with endocrine therapy in OS (*p* = 0.030) and was close to significance for BCSS (*p* = 0.056). TSR did not predict outcome in untreated luminal tumours (OS *p* = 0.209 and BCCS *p* = 0.059).

## 3. Discussion

TSR was first described as a prognostic factor in colorectal carcinoma in 2007 [28] and it has subsequently been assessed in most solid tumours (reviewed in [8]) with most tumour types showing an association of high stroma (low TSR) with poor outcome. In breast cancer, several studies have consistently demonstrated high stroma to be unfavourable for outcome in TNBC and in most studies of ER+ cancer [10,11,12,22,29]. However, in luminal ER+ disease, one other study also found ER+ cancer (118 females and 62 males) with high stroma (low TSR) to be associated with improved outcome [13]. No association of TSR with outcome was found in a small group of inflammatory breast cancers [23]. The rationale for our study approach was to specifically address the hypothesis that there may be inherent differences between the stroma of TNBC and luminal cancer and hence we selected subtype-specific cut points to perform our analysis. Of significance, our approach confirms the poor independent prognostic significance of high stroma in TNBC, but also supports its favourable prognostic significance in ER+ cancer.

One of the key points to consider in this study is the variation in the methods applied to assess TSR. The majority of studies in breast have been performed by a prominent single group of investigators who have made a major contribution to this area and developed a standardised assessment method [10,11,22,28]. Their method reviewed whole tumour sections and identified a single region of interest (ROI) at ×10 magnification, containing the highest amount of stroma, equivalent to an area of 3.1 mm^2^. Additionally, the ROI selected must have tumour cells present on all sides of the target region field of view. Stroma is then scored incrementally in 10% intervals with a 50% cut-point for high or low. The only other study which found a favourable association of high stroma and outcome in ER+ cancer [13] used a computer-assisted selection of two areas, each of 9 mm^2^ from the leading edge and non-leading edge, using a digital slide image [13]. Random point counting assessed 300 spots per ROI as tumour, stroma or uninformative. Cut point determination was assessed by performing multiple log-rank tests for outcome to obtain the lowest p value, resulting in 49% being applied to the cohort. Although both methods have similar cut points, the areas sampled and the methods for ROI selection are different. Using our TMA approach of random sampling from the tumour periphery of 3 × 1 mm cores (an area equivalent to 2.35 mm^2^ for assessment), also supported the finding of high stroma as an adverse prognostic finding in TNBC. Interestingly our TSR cut-point of 2 is equivalent to approximately 66% tumour and 33% stroma. This stromal value cut-point of 33% is slightly less than the 50% stromal visual cut-point of the other major studies. The 17% difference between human visual and computer assessment in cut-point suggests there may be a common discrepancy between human versus computer when assessing a tumour pathology image. A similar degree of discrepancy between human and image analysis stromal volume assessment was also described between the cut-point for the convolutional neural network (CNN) based analysis of colorectal cancer [24] CNN cutpoint 65.47% versus 50% visual assessment. Similar CNN methodology was also recently used to combine the assessment of primary tumour and lymph nodal metastases TSR, which was also of prognostic value [30].

The long follow-up in our cohort also provides additional clinical interest for ER+ disease which is characterised by frequent late recurrences compared to TNBC which recurs mostly within 5–6 years of diagnosis. The selection of TMAs, in this regard, provides interesting data which support the association of high stroma and poor outcome in TNBC but good outcome in ER+ disease. Our study offers an alternative methodology using TMAs and automated image analysis and machine learning algorithms, which provides rapid, objective, quantitative area estimation, suitable for application to large clinical trial cohorts with efficient rapid throughput of data. Additionally, it may also be of benefit for cases close to a visual cut-point method requiring consensus review.

Whilst the QuPath algorithm used in our study performed reasonably well, it required continuous supervision by a Pathologist, which is one of the main limitations of this study. The segmentation of more complex visual features with accurate classification requires higher capabilities provided by deep learning CNN to distinguish between subtle shades of colour and textured qualitative difference in staining and distinction of TILs [31]. Therefore, whether our results are influenced by errors of algorithm classification remains to be seen. Given the variation in cut point and methods used in existing publications, further studies potentially using AI will likely see image analysis methods further developed over time to determine organ and possibly tumour subtype-specific measurements and cut points. It could also be argued that the use of TMAs as a sample of a whole slide image (WSI) is also a limiting factor. However, our data appear to suggest that a small sample is a prognostically relevant surrogate of TSR for a WSI.

In terms of biology, the assessment of TSR per se is only a simplified ratio which quantifies tumour cellularity and stroma prominence. The stromal TME is a complex space, comprising multiple cell types. Further support for the presence of low TSR (i.e., abundant stroma) as a predictor of good prognosis in ER+ cancer is provided through our findings of high correlation of low TSR with low histological grade and type, i.e., ILC and tubular carcinomas suggesting TSR in ER+ cancer may be a surrogate for histological type. Additionally, high stromal proteomics signatures in ER+ cancer also defined good prognosis in two independent datasets (TCGA and MD Anderson) [32].

A key question is how the stroma of breast cancer may contribute to outcome. TILs density appears to correlate with TSR, most notably for TNBC, which tend to be immune “hot” tumours compared to immune “cold/desert” luminal tumours. In our further exploratory analyses in TNBC, low sTILs and low TSR (i.e., high stroma) had the worst prognosis compared to the best group of high TILs and high TSR (i.e., low stroma). This supports the findings of high stroma and low immune status, characterised by low HLA-class I in TNBC, which had a 35% 10-year recurrence-free period versus 73% for TNBC expressing HLA-class I [33]. The presence of sTILs and stromal predominance are features that may be of value in our understanding of radiomics prediction of response to neoadjuvant chemotherapy in TNBC and in the assessment of subtype prediction using MRI and contrast-enhanced spectral mammography [34,35,36].

Detailed single cell RNA-seq (scRNA-seq) data are now emerging regarding the heterogenous nature of the stromal cell population, which provides cellular resolution not previously available from bulk sequencing studies [37,38]. One study of human TNBC identified two distinct populations of CAFs: myofibroblastic (myCAFs) and inflammatory (iCAFs) and two populations of peri-vascular-like cells (PVL) [38]. Through analysis of large RNA-Seq datasets, the authors showed that PVL cells were associated with TILs exclusion, perhaps explaining our observation of a positive correlation between TSR and TIL. How these cell populations spatially relate to cancer cells and immune cells to influence immunosuppression, tumour growth and treatment response via complex cellular “cross-talk” is an area of active interest with potential for the development of new stromal targeted therapeutics and biomarkers.

Numerous markers have been used to define CAFs subtypes in human tissue but most of these are not specific to CAFs alone and, often, dual expression is required to delineate their phenotype (e.g., α-SMA, FAP, FSP-1, PDGFR, CD90, PDPN, S100A4). Notably, CD10 + GPR77 + CAFs were found to promote cancer stemness and chemoresistance in breast cancer with restoration of chemosensitivity using targeted therapeutic antibody in PDX models [16]. Similarly, Hedgehog (Hh) ligand, derived from TNBC cancer cells in PDX models, is capable of reprograming CAFs to induce chemoresistance with the production of fibrillary collagen, which can be reversed using Smoothened inhibitors (SMOi) to induce chemosensitivity [15]. Fibrillary collagen is a known breast cancer risk factor [39] and recent CNN analyses of the orientation of collagen fibres in H&E sections of breast tumours [40], and specifically in ER+ disease, also correlates with outcome [41]. CD146 + CAFs have also been associated with endocrine responsiveness ER+ cancer [7].

## 4. Materials and Methods

### 4.1. Clinical Cohorts

#### 4.1.1. Luminal Cohort

**Figure 5:**
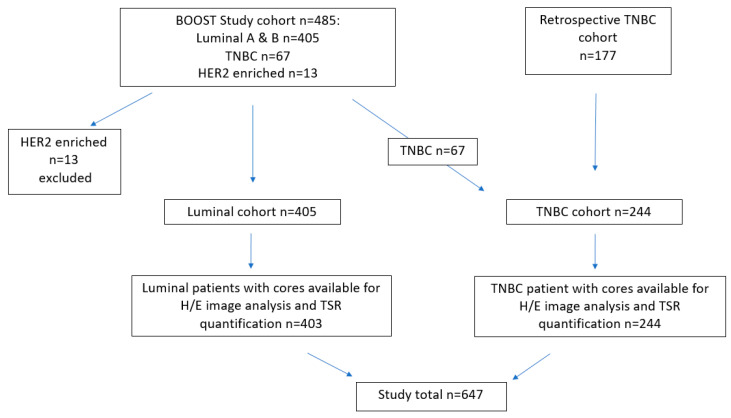
CONSORT flow diagram of the composition of the luminal and triple negative breast cancer (TNBC) samples used in the study.

The patient cohort was derived primarily from the St George Breast Boost randomised radiotherapy clinical trial (1996–2003, *n* = 485) supplemented by a further group of retrospective TNBC cases identified from the archive of the Department of Anatomical Pathology at St George Hospital, Kogarah, NSW, Australia (*n* = 177). The St George Boost cohort is well characterised, previously published and summarised as follows [42,43,44,45]: all patients received wide local excision with whole breast irradiation (45Gy with boost; 50Gy if no boost in 25 fractions), randomised to a cavity boost (16Gy in 8 fractions) or not (ClinicalTrials.gov NCT00138814). This trial recruited all subtypes of invasive breast cancer (stages Tis-3, N0-3, M0). The TMA cohort derived from this trial (from a total trial cohort of *n* = 688) comprised 405 ER+ invasive luminal tumours (309 luminal A and 96 luminal B) which formed the luminal cohort with a median follow-up of 16.4 years (range 0.1–21.3) subtyped using immunohistochemistry criteria [46]. There were only 13 HER2 enriched cases which was too small a group to be meaningful for further analyses. The 67 TNBC from this trial cohort were subsequently added to a cohort of retrospective TNBC tumours, created to further examine disease associations for this study, as outlined in the CONSORT flow diagram (Figure 5). The REMARK guidelines for biomarker assessment were followed [47].

#### 4.1.2. TNBC Cohort

A review of Oncology databases at St George Hospital were used to identify cases of TNBC diagnosed from 2004–2018. From this pool, 177 cases were included in this cohort which had enough tumour remaining in the formalin fixed paraffin embedded (FFPE) blocks. To this group were added 67 TNBC cases present in the Boost cohort described above, creating a total cohort size of n = 244. All TNBC cases were negative for ER, PR (<1%) and HER2 (by IHC and SISH). Tumour average size was 25.9 mm (range 7–120 mm), average age at diagnosis 58 years, median follow-up 4.3 years (range 0.02–16.3), 232 cases (95%) were grade 3, 111 (45%) had high stromal tumour infiltrating lymphocytes (sTILS > 30%), 90.5% were invasive ductal carcinoma of no special type with 7% metaplastic carcinoma, 2.5% other (apocrine, micropapillary) with nodal positivity in 85 cases (35%). There were 71 deaths, 48 of which were breast cancer related. All cases were scored for sTILs on whole tumour sections by an experienced breast Pathologist using standardised criteria [48]. Ethics approval was provided by SESLHD Human Research Ethics Committee at the Prince of Wales Hospital, Sydney (Boost: HREC 96/16 and TNBC: HREC 2018/ETH00138).

### 4.2. TMA Construction

All TMAs were constructed using a Beecher Manual Arrayer MTA-1 (Beecher Instruments, Inc., Sun Prairie, WI, USA). All donor tumours were reviewed on a Haematoxylin and Eosin (H&E) slide and appropriate areas for sampling of the block were marked up by a breast Pathologist. Then, 3 × 1 mm cores were sampled from the periphery of each tumour block. Donor cores were placed within the recipient block in a predetermined layout to distribute the cores evenly across each array. Paraffin sections were cut at 4µm onto Superfrost™ glass slides (ThermoFisher Scientific, Waltham, MA, USA) and stained with H&E using a Leica automated staining machine (Leica Biosystems, Nussloch, Germany) in the Department of Anatomical Pathology, NSW Health Pathology, St George Hospital, Kogarah, Australia.

### 4.3. Digital Scanning

H&E-stained sections of all TMAs were digitally scanned using the Ventana DP200 digital scanner (Roche Diagnostic, Tucson, AZ, USA) at ×400 magnification (0.25 µm per pixel) and stored as .TIF files. Following scanning, all TMA cores were quality checked by a Pathologist to ensure all regions were in focus without blurring prior to analysis.

#### Image Analysis

All digital analyses were performed using QuPath [25] v0.2.1 (https://qupath.github.io/), an open source digital image analysis software platform with built in trainable machine learning image analysis algorithms. All TMA files and corresponding TMA maps (.csv files) were imported using the TMA module and orientated appropriately. All tissue core identification number labelling outputs, using the TMA de-arrayer, were checked prior to any analyses. Further quality steps were taken to review cores with any folded, blurred, missing or imperfect morphology were excluded from analyses. Pre-processing was then applied by to allow colour vector deconvolution and sampling of red-green-blue (RGB) values to define colour channels. Representative training areas were then selected to segment tumour epithelium (red) and stroma (green) with the background white (and also fat lobules) set to “ignore”. Algorithm training was applied to produce the optimal tissue classification with additional training regions added as required to improve the accuracy of classification of epithelium and stroma to minimise detection of peri-tumoral TILs nuclei as epithelia. The pixel classifier was then applied to all TMAs in the study sequentially. Data output from QuPath, using the TMA data reviewer, provided the area of tumour epithelium and stroma separately in µm^2^ per TMA core and as a percentage value. We used the mean value for tumour and stroma from 3 cores per patient tumour (total area 2.35 mm^2^). The tumour stroma ratio (TSR) was then calculated by dividing the epithelial area by the stromal area.

### 4.4. Statistical Analyses

The association of clinicopathological features and TSR was performed using a X^2^ test. Time to event outcomes (OS, BCSS) were assessed using Cox proportional hazards for univariate and multivariate analyses, where *p* < 0.05 was considered significant. Overall survival (OS) was defined as the time from randomization to death from any cause. Breast cancer specific survival (BCSS) was defined as death directly attributable to breast cancer. Survival estimates were displayed by Kaplan–Meier analyses. All analyses were performed by an experienced statistician using STATA V11 (StataCorp LLC, College Station, TX, USA).

## 5. Conclusions

In summary, automated image analysis estimation of TSR using TMAs validates the findings of high stroma as an independent poor prognostic factor in TNBC. In contrast to most of the published literature in ER+ disease, we found that high stroma is a favourable prognostic feature which likely reflects lower grade and favourable histological types. Further investigation of the CAFs phenotype could potentially refine the incorporation of these data into future clinical trials for breast cancer.

## Figures and Tables

**Table 1:** Univariate and multivariate analysis for overall survival (OS) and breast cancer specific survival (BCSS) in triple negative breast cancer (TNBC). Abbreviations: n, sample number; HR, hazard ratio; CI, confidence interval; *p*, probability value).

Overall Survival			Univariate			Multivariable (*n* = 238, Events *n* = 66)		
Variables		n	HR	95%CI	*p*	HR	95%CI	*p*
TSR	≤2 vs. >2	151 vs. 93	1.54	0.93–2.55	0.093	1.90	1.10–3.29	0.021
TILs	≤30 vs. >30	133 vs. 111	1.66	1.02–2.70	0.040			
Age	≤55 vs. >55	105 vs. 138	0.48	0.29–0.80	0.004	0.41	0.24–0.70	0.001
Size	≤20 vs. >20	113 vs. 129	0.54	0.33–0.89	0.016	0.51	0.30–0.87	0.014
Grade	1,2 vs. 3	12 vs. 232	1.46	0.63–3.38	0.379			
LN	neg vs. pos	156 vs. 85	0.43	0.27–0.70	0.001	0.46	0.28–0.76	0.003
Chemo	yes vs. no	174 vs. 58	0.49	0.30–0.81	0.006			
**Breast Cancer Specific Survival**			**Univariate**			**Multivariable** **(*n* = 238, Events *n* = 46)**		
**Variables**		**n**	**HR**	**95%CI**	***p***	**HR**	**95%CI**	***p***
TSR	≤2 vs. >2	151 vs. 93	2.34	1.19–4.59	0.014	2.64	1.31–5.35	0.007
TILs	≤30 vs. >30	133 vs. 111	1.89	1.03–3.44	0.038			
Age	≤55 vs. >55	105 vs. 138	0.50	0.27–0.91	0.022	0.43	0.23–0.80	0.008
Size	≤20 vs. >20	113 vs. 129	0.45	0.24–0.85	0.013	0.46	0.24–0.89	0.020
Grade	1,2 vs. 3	12 vs. 232	1.48	0.53–4.14	0.453			
LN	neg vs. pos	156 vs. 85	0.31	0.17–0.55	<0.001	0.32	0.18–0.59	<0.001
Chemo	yes vs. no	174 vs. 58	0.69	0.37–1.30	0.253			

**Table 2:** Univariate and multivariate analysis for OS and BCSS in luminal ER+ breast cancer. Abbreviations: n, sample number; HR, hazard ratio; CI, confidence interval; p, probability value).

Overall Survival			Univariate			Multivariable (*n* = 402, Events *n* = 149)		
Variables		n	HR	95%CI	*p*	HR	95%CI	*p*
TSR	≤0.74 vs. >0.74	202 vs. 201	0.65	0.47–0.90	0.010	0.56	0.40–0.77	0.001
TILs	≤10 vs. >10	325 vs. 78	1.71	1.06–2.77	0.029	1.72	1.06–2.81	0.030
Age	≤55 vs. >55	139 vs. 264	0.34	0.22–0.52	<0.001	0.31	0.20–0.48	<0.001
Size	≤20 vs. >20	294 vs. 108	0.70	0.50–0.99	0.046	0.66	0.47–0.94	0.021
Grade	1,2 vs. 3	321 vs. 80	0.98	0.65–1.47	0.926			
LN	neg vs. pos	282 vs. 121	0.71	0.51–0.99	0.043			
Chemo	yes vs. no	63 vs. 340	0.69	0.42–1.15	0.153			
Horm	yes vs. no	206 vs. 196	1.21	0.87–1.67	0.257			
**Breast Cancer Specific Survival**			**Univariate**			**Multivariable** **(*n* = 400, Events *n* = 39)**		
**Variables**		**n**	**HR**	**95%CI**	***p***	**HR**	**95%CI**	***p***
TSR	≤0.74 vs. >0.74	202 vs. 201	0.39	0.20–0.78	0.007			
TILs	≤10 vs. >10	325 vs. 78	0.93	0.43–2.03	0.860			
Age	≤55 vs. >55	139 s 264	1.66	0.89–3.12	0.113			
Size	≤20 vs. >20	294 vs. 108	0.29	0.16–0.55	<0.001	0.45	0.23–0.87	0.021
Grade	1,2 vs. 3	321 vs. 80	0.30	0.16–0.57	<0.001	0.38	0.20–0.72	0.003
LN	neg vs. pos	282 vs. 121	0.20	0.11–0.40	<0.001	0.19	0.09–0.41	<0.001
Chemo	yes vs. no	63 vs. 340	3.46	1.82–6.60	<0.001			
Horm	yes vs. no	206 vs. 196	1.11	0.59–2.09	0.742	0.45	0.23–0.88	0.020

## Supplementary Materials

The following are available online at https://www.mdpi.com/2072-6694/12/12/3749/s1, Figure S1: The distribution of tumour stroma ratios within the study cohorts, Table S1: Association of tumour stroma ratio (TSR) with clinico-pathological variables using the Χ^2^ test. * TILs cut point ≤ 30% for TNBC and ≤10% for luminal. Meta: metaplastic carcinoma, Table S2: Univariate and multivariate analysis for OS and BCSS in TNBC.
