# Supplementary material for: Tumour Stroma Ratio Assessment Using Digital Image Analysis Predicts Survival in Triple Negative and Luminal Breast Cancer
Source: Cancers (Basel). 2020 Dec 13;12(12):3749. doi: 10.3390/cancers12123749 (PMC7764351; doi:10.3390/cancers12123749)
Supplement: Supplementary file 1 [file cancers-12-03749-s001.pdf]

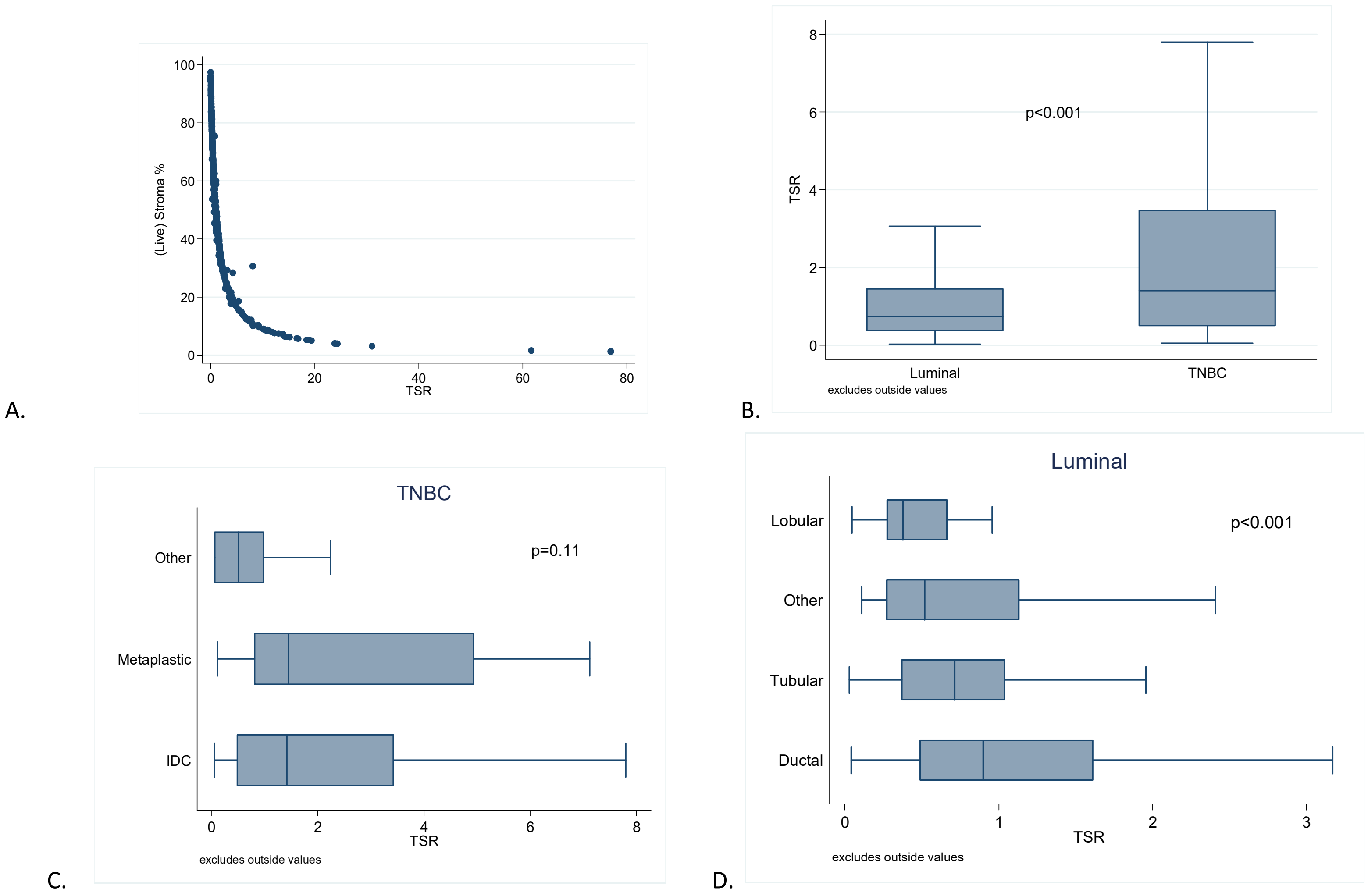

Supplementary figure 1. The distribution of tumour stroma ratios within the study cohorts. A: distribution of TSR scores versus stromal percentage for the whole cohort. B. Box plot to illustrate the distribution of TSR scores between luminal and TNBC. C: TSR scores for TNBC ; D TSR scores for luminal histological types. (C: TNBC other includes apocrine, micropapillary; D: Luminal other: includes micropapillary, mixed, mucinous).

	TNBC							Luminal						
		TSR (cut 2)			TILs (cut 30)				TSR (cut 0.74)			TILs Cut (10)		
	**Total**	**low**	**high**	**X^2 p value**	**low**	**high**	**X^2 p value**	**Total**	**low**	**high**	**X^2 p value**	**low**	**high**	**X^2 p value**
**Age**														
**≤55**	105(43)	55(36)	50(54)	7.5	55(42)	50(45)	0.28	139 (34)	60(30)	79(39)	4.1	104(32)	35(44)	4.61
**>55**	138(57)	96(64)	42(46)	0.006	77(58)	61(55)	0.596	264(66)	142(70)	122(61)	0.043	221(68)	43(55)	0.032
**Size**														
**≤20mm**	113(47)	69(46)	44(48)	0.08	58(44)	55(50)	0.67	294(73)	158(78)	136(68)	5.3	237(73)	57(74)	0.04
**>20mm**	129(53)	81(54)	48(52)	0.782	73(56)	56(50)	0.412	108(27)	44(22)	64(32)	0.021	88(27)	20(26)	0.844
**Grade**														
**1 & 2**	12(5)	10(7)	2(2)	2.46	11(8)	1(1)	7.03	321(80)	179(89)	142(71)	18.7	273(84)	48(62)	18.7
**3**	232(95)	141(93)	91(98)	0.117	122(92)	110(99)	0.008	80(20)	23(11)	57(29)	<0.001	51(16)	29(38)	<0.001
**LN status**														
**Neg**	156(65)	94(63)	62(68)	0.74	87(66)	69(63)	0.35	282(70)	151(75)	131(65)	4.4	228(70)	54(69)	0.03
**Pos**	85(35)	56(37)	29(32)	0.389	44(34)	41(37)	0.551	121(30)	51(25)	70(35)	0.036	97(30)	24(31)	0.873
**TILs (*)**														
**≤30/ ≤10**	133(55)	92(61)	41(44)	6.58				325(81)	180(89)	145(72)	18.59			
**>30/ >10**	111(45)	59(39)	52(56)	0.010				78(19)	22(11)	56(28)	<0.001			
**Hist type**														
**Ductal**	221(91)	136(90)	85(91)	1.25	114(86)	107(96)	9.08							
**Meta**	17(7)	10(7)	7(8)	0.536	13(10)	4(4)	0.011							
**Other**	6(2)	5(3)	1(1)		6(4)	0(0)								
**Ductal**								286(71)	123(61)	163(81)	23.88	219(67)	67(86)	10.5
**Lobular**								31(8)	25(12)	6(3)	<0.001	28(9)	3(4)	0.015
**Tubular**								29(7)	16(8)	13(7)		26(8)	3(4)	
**Other**								57(14)	38(19)	19(9)		52(16)	5(6)	

Supplementary table 1. Association of tumour stroma ratio (TSR) with clinico-pathological variables using the X^2 test.*TILs cut point ≤30% for TNBC and ≤10% for luminal. Meta: metaplastic carcinoma.

**Overall Survival**		**Univariate**			**Multivariable (n=238, events n= 66)**		
**Variables**		**HR**	**95%CI**	**p**	**HR**	**95%CI**	**p**
TSR/TILS 4 group (vs low/low)							
low/high	59 vs 92	0.53	0.29 - 0.99	0.047	0.61	0.32 – 1.14	0.119
high/low	41 vs 92	0.56	0.28 – 1.13	0.107	0.44	0.18 – 1.06	0.067
high/high	52 vs 92	0.49	0.25 – 0.94	0.033	0.45	0.23 – 0.87	0.018
Age ≤55 vs >55	105 vs 138	0.48	0.29 – 0.80	0.004	0.42	0.25 – 0.72	0.001
Size ≤20 vs >20	113 vs 129	0.54	0.33 – 0.89	0.016	0.53	0.31 – 0.90	0.019
Grade 1,2 vs 3	12 vs 232	1.46	0.63 – 3.38	0.379			
LN neg vs pos	156 vs 85	0.43	0.27 – 0.70	0.001	0.47	0.28 – 0.79	0.004
Chemo yes vs no	174 vs 58	0.49	0.30 – 0.81	0.006			
**Breast Cancer Specific Survival**		**Univariate**			**Multivariable (n=238, events n= 46)**		
**Variables**	**n**	**HR**	**95%CI**	**p**	**HR**	**95%CI**	**p**
TSR/TILS 4 group (vs low/low)							
low/high	59 vs 92	0.45	0.21 – 0.95	0.036	0.53	0.25 – 1.13	0.098
high/low	41 vs 92	0.29	0.10 – 0.82	0.019	0.31	0.09 – 1.04	0.057
high/high	52 vs 92	0.36	0.16 – 0.82	0.016	0.31	0.13 – 0.72	0.006
Age ≤55 vs >55	105 vs 138	0.50	0.27 – 0.91	0.022	0.44	0.23 – 0.83	0.012
Size ≤20 vs >20	113 vs 129	0.45	0.24 – 0.85	0.013	0.48	0.25 – 0.94	0.031
Grade 1,2 vs 3	12 vs 232	1.48	0.53 – 4.14	0.453			
LN neg vs pos	156 vs 85	0.31	0.17 – 0.55	<0.001	0.33	0.18 – 0.62	0.001
Chemo yes vs no	174 vs 58	0.69	0.37 – 1.30	0.253			

Supplementary Table 2. Univariate and multivariate analysis for OS and BCSS in TNBC. Abbreviations: n, sample number; HR, hazard ratio; CI, confidence interval; p, probability value).
